# Assessing the Quality of Teleconsultations in a Store-And-Forward Telemedicine Network – Long-Term Monitoring Taking into Account Differences between Cases

**DOI:** 10.3389/fpubh.2014.00211

**Published:** 2014-10-28

**Authors:** Richard Wootton, Joanne Liu, Laurent Bonnardot

**Affiliations:** ^1^Norwegian Centre for Integrated Care and Telemedicine, University Hospital of North Norway, Tromsø, Norway; ^2^Faculty of Health Sciences, University of Tromsø, Tromsø, Norway; ^3^Médecins Sans Frontières International, Geneva, Switzerland; ^4^Department of Medical Ethics and Legal Medicine, EA 4569, Paris Descartes University, Paris, France; ^5^Fondation Médecins Sans Frontières, Paris, France

**Keywords:** telemedicine, telehealth, quality assurance, quality control, LMICs

## Abstract

We have previously proposed a method for assessing the quality of individual teleconsultation cases; this paper proposes an additional step to allow the long-term monitoring of quality. The basic scenario is a teleconsultation system (aka an e-referral system or a tele-expertise system) where the referrer posts a question about a clinical case, the question is relayed to an appropriate expert, and the chosen expert provides an answer. The people running this system want assurances that it is stable, i.e., they want routine quality assurance information about the “output” from the “process.” This requires two things. It needs a method of assessing the quality of individual patient consultations. And it needs a method for taking into account differences between patients, so that these quality assessments can be compared longitudinally. Building on the previously proposed methodology, the present paper proposes two techniques for measuring the difficulty posed by a particular teleconsultation. The first is an indirect method, similar to a willingness to pay economic estimation. The second is a direct method. Using these two methods with real data from a telemedicine network showed that the first method was feasible, but did not produce useful results in a pilot trial. The second method, while more laborious, was also feasible and did produce useful results. Thus, when output quality is measured, an allowance can be made for the characteristics of the case submitted. This means that fluctuations in output quality can be attributed to variations in the process (network) or to variations in the raw materials (queries submitted to the network). Long-term quality assurance should assist those providing telemedicine services in low-resource settings to ensure that the services are operated effectively and efficiently, despite the constraints and complexities of the environment.

## Introduction

Telemedicine has been used for many years to support doctors working in low-resource settings. Sometimes real-time telemedicine is used, for example, video links between a doctor in the field and a specialist, but more commonly store-and-forward telemedicine is employed, because it is cheaper and easier to organize. Médecins Sans Frontières (MSF), a non-governmental humanitarian medical organization, has used both approaches (1–3). The store-and-forward telemedicine network, which it currently operates can be viewed as a logical development of its work, where doctors working with scarce resources in remote settings can obtain specialist medical advice for specific patients.

As telemedicine matures and becomes adopted as a routine method of healthcare delivery, there is an obligation to implement quality assurance/improvement activities. All provider organizations need to demonstrate that they are providing high-quality care via validated and controlled tools.

### Operation of a telemedicine network

A store-and-forward telemedicine network of the type under discussion provides “tele-expertise” to doctors in the field. These field users can submit clinical queries to the network, and based on some internal mechanism (not relevant here), the query is sent to an appropriate expert for reply. In other words, the telemedicine network can be regarded as a “black box” (4), which accepts an input, carries out some action, and produces an output. That is, a clinical query is put into the black box, it is processed in some way, and an expert answer comes out. In the longer term, this can be viewed as a production process, similar to the manufacture of goods in factory: raw materials arrive, they are processed, and the resulting goods represent the output.

### Statement of the problem

If a telemedicine network is viewed as a black box, then industrial methods for controlling the process become relevant. In industrial production processes, it is usually desirable to measure the quality of the output and ensure that this meets some target value. To do this, the output from a production run is sampled intermittently and judged against a suitable standard. For example, the output from a factory bottling wine might be judged by weighing the bottles to confirm that they had been filled satisfactorily. Let us suppose that the target weight for the contents of the bottles is 700 g. A sample bottle can be weighed when empty and again after it has been filled, allowing the weight of the contents to be determined accurately. To carry out quality control, bottles will be sampled regularly and the content weights will be plotted on a process control chart. The filling process will be considered satisfactory if the average content weight is sufficiently close to the target and there are no indications that the average weight is drifting either up or down. Conventional process control therefore depends on a method for measuring the output achieved and a comparison with a target (the desired output).

Now consider the quality of teleconsultations, selected from the “output” of a telemedicine network. Again, the process operators (i.e., the people responsible for running the network) may wish to know that the process is stable. That is, they want confirmation that the quality of the teleconsultations is satisfactory and that the average quality is not declining. (They may not object if the average quality is increasing, of course.)

In conventional process control, the output from the telemedicine network would be measured, and compared with a predetermined target value. We have previously described a method for measuring output (5), but it is not straightforward to define a target value for a telemedicine network. A quantitative description of the quality of an ideal teleconsultation is not possible in the current state of our knowledge. Instead, for process control purposes, we propose the measurement of the quality of the *input* to the process, so that observed fluctuations in output quality can be attributed to variations in the process (network) or to variations in the raw materials (queries submitted to the network).

*Measuring the output*. Our previous paper (5) sets out a method by which output quality can be assessed. A panel of observers makes a judgment about whether various aspects of the teleconsultation are considered satisfactory or not. These assessments are made without an explicit quality standard – they are actually based on the judgment made by each observer, which are aggregated to form a panel view. Thus, the assessment relates to the patient in question, but there is no way of accounting for the fact that patients differ. In other words, these are assessments of quality achieved in individual cases, but in the absence of a target (the desired quality), it is very difficult to monitor any long-term drift in quality.
*Measuring the input*. This requires a method for assessing the submitted queries and the resources available to the network for answering them. In real life, the “production setting” differs each time a query is submitted because patients are different from one another. Sometimes, the telemedicine question may be straightforward, e.g., “*here is a chest X-ray; does this show TB?*” Sometimes, the telemedicine question may be very complex, e.g., “*here is some clinical history; we don’t know what the diagnosis is and we don’t know how to manage the patient; can you help?*” The production setting can also be made more difficult by the non-availability of specific experts required for certain cases. Thus, if the difficulty of the cases is not taken into account, it is not possible to assess output quality longitudinally.

Process control in a telemedicine network therefore requires two things. It needs a method of assessing the quality of the individual patient consultations, which are produced. And it needs a method for taking into account differences between cases, so that these quality assessments can be compared. To allow for the differences between patients, we need a method of measuring the “difficulty” of the question being posed to the teleconsultation network.

An analogy is a process, which produces a food – say, airline meals – from a raw material. The raw materials (ingredients) vary from batch to batch, but the process operators require the product to be as consistent as possible. So in some batches, much more skill is required (i.e., if the case in question is “difficult”). In the commercial kitchen example, this might mean preparing the food at a different temperature and/or for a different time. There may be instances where such a poor batch of ingredients is supplied that the quality of the product suffers. Quality monitoring would then show that this batch was of lower quality, and would also reveal the reason why: the “case” was extremely difficult because of substandard raw material. In other instances, a poor batch of ingredients might be supplied, yet the skill of the production operatives (chefs) might ensure that the output quality was normal.

Thus, the problem addressed in the present work is the development of a method that can be used by the people responsible for running a telemedicine network to monitor its operation with the aim of determining whether the process is stable and whether the quality of the teleconsultations is being maintained. This requires a method for taking into account differences between cases, so that these quality assessments can be compared. As far as we are aware, there has been no previous work on this subject.

### Difficulty of the teleconsultation case

The difficulty of a submitted case will be partly dependent on the clinical complexity of the patient. (Only partly, because we could have a complex question about a simple clinical problem or vice versa). In fact, the difficulty of the case depends on four main factors:

(1) the description of the problem
(2) the complexity of the patient
(3) the availability of network resources for providing an answer
(4) the availability of resources for implementing the advice (i.e., for providing treatment).

That is, from the point of view of the telemedicine network that receives a new case, it may be difficult to provide an answer because the problem is badly described, because the patient has a very complex illness, because the network does not have the right expert available to respond, or because the case is being managed in a remote hospital where treatment options are likely to be limited. Some or all of these difficulties may be present in any given case. Furthermore, each of these factors depends on various sub-factors:

(1) the description of the problem depends on how well formulated the question is, and how much information is provided about the patient (e.g., whether satisfactory images were supplied with the case, if appropriate).
(2) the complexity of the patient can be measured in different ways. One accepted approach is to determine the severity of the illness; the presence of multiple co-occurring medical conditions; the difficulty in determining an accurate diagnosis and/or management plan; the degree of impairment or disability that results from the medical condition; the level of need for comprehensive care management (6). That is, health care complexity reflects not only medical or biological complexity but also the management of the condition, the context of the condition, the interactions between the person and the provider or the service, and the broader environment (7).
(3) the availability of network resources for providing an answer depends on having suitable case-coordinators available and on the availability of whatever specialists/subspecialists are needed to provide a definitive response.
(4) the availability of resources for providing treatment depends on the size of hospital (a proxy for the resources available locally), local facilities and their capacity, and the ease with which a referral could be made elsewhere for specialist treatment if required.

The situation is summarized in Table 1. Thus, if we measure the outputs from a telemedicine network and find that output quality is declining, we want to be able to distinguish between a problem with the production process itself and a problem with the raw material (i.e., a more complex patient or a poorly described question from the referrer).

**Table 1:** **Summary of the factors affecting the difficulty of a case presented to a telemedicine network**.

Main factor	Constituent factors
1. Description of the *problem*	1a. Formulation of the question
	1b. Information provided (including images and their quality)
2. Intrinsic *difficulty* (complexity of the patient)	2a. Severity of the illness
	2b. Co-occurring medical conditions
	2c. Difficulty in determining an accurate diagnosis
	2d. Degree of impairment or disability
	2e. Need for comprehensive care management
3. Network resource available for providing the *answer*	3a. Availability of care-coordinator resource (if manual case allocation is being used)
	3b. Availability of required specialists/subspecialists
4. Resource available for providing recommended *treatment*	4a. Treatment resources available locally
	4b. Possibility of transfer for specialist treatment elsewhere, if required

### Objective

The objective of the present work was to develop a method for determining the difficulty of a case being submitted for teleconsultation, able to take into account the differences between patients.

## Methods

We propose two methods for determining the difficulty of the case submitted to a teleconsultation network. The first is indirect, and the second is direct. The feasibility of each method was trialed using data from an operational telemedicine network. Ethics permission was not required, because patient consent had been obtained prior to submitting each case and the work concerned the retrospective review of anonymized data conducted by the organization’s staff in accordance with its research policies.

### First method – indirect assessment

#### Background – willingness to pay

In health economics, an established technique for estimating the value of a product is to find out people’s willingness to pay (WTP) for it. Technically, WTP is the maximum amount that a person is willing to sacrifice to procure a good or to avoid something undesirable. This is usually established by surveying a group of consumers who are asked questions such as, “Would you purchase this product if it were offered at a price of X?” If this price differs between the consumers surveyed, then it is possible to make a good estimate of the sample’s collective WTP a particular price.

Willingness to pay surveys have been used in medicine generally and in telemedicine specifically. For example, in one of the earliest telemedicine studies, Tsuji et al. (8) surveyed users of a home telemonitoring system in Japan; the best estimate of the WTP was ¥4519 (approximately US$37) per user per month (8). Bergmo and Wangberg surveyed patients in a Norwegian general practice to investigate their WTP for teleconsultations. Approximately half of the patients were willing to pay for electronic contact with their GP (9). Bradford et al. investigated the willingness of patients with chronic heart failure to pay for access to medical care via telemedicine, as an alternative to traveling to the physician’s office. They found that 55% of the patients surveyed would be willing to pay $20 to access telemedicine instead of traveling to the physician’s office, for at least some of their care (10).

#### Estimation of case difficulty

We have previously proposed a method for assessing the quality of a teleconsultation, which requires a panel of observers to answer questions about a selected case. The method provides indices (scores) relating to different aspects of quality (5). The present proposal extends this methodology to take account of the difficulty posed by an individual case. This is estimated by a consensus among those reviewing the case, as follows.

Suppose four panel members review a case, answer the value questions independently, but are not told what the overall value (score) of their responses is. Then they are asked a final question: “Considering the teleconsultation as a whole, do you think the quality (value) was sufficiently good in the circumstances? In other words, quality can always be made higher, but was it good enough?”

That is, the final question takes into account the specificity of the environment and its variability. Their individual answers to this question might be:

Y,Y,N,N

If the corresponding quality scores (i.e., each member’s assessment of the value achieved, on a Likert scale from 0 to 10) are computed, these might turn out to be:

9.1,8.5,6.9,7.5

From the first two responses, we know that the score of 9.1 was considered high enough (by panel member 1), but that a score of 8.5 was also considered high enough (by member 2). That is, 8.5 represents the upper bound on the quality required.

From the other answers, we know that 6.9 was not considered high enough (by member 3) and that 7.5 was not considered high enough either (by member 4). That is, the lower bound lies *just above* 7.5. In the scoring system under discussion, a precision of more than 1% would not be meaningful. Thus, a lower bound lying just above 7.5 can be taken as a value of 7.6.

Therefore, in this example, the value can be estimated to lie in the range 7.6–8.5. This represents a consensus view about the quality of the teleconsultation, taking into account the circumstances of the case, such as whether the clinical question was very complex.

In establishing the consensus view of the panel, there are three possible sets of answers:

(A) Some panel members answer that their individual estimate was sufficient and some answer that it was not.
(B) All panel members answer that their estimate was sufficient.
(C) All panel members answer that their estimate was not sufficient.

**Figure 1:**
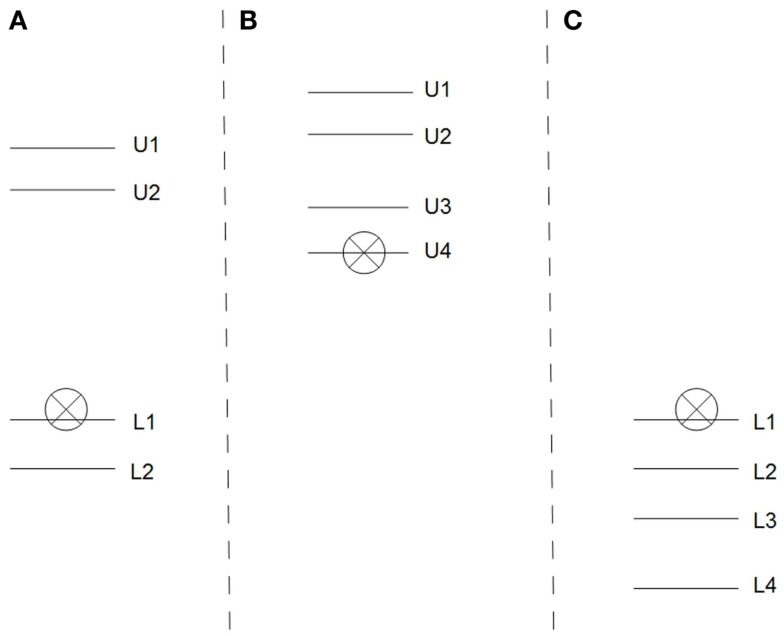
**Indirect assessment: three scenarios**. The subscripts indicate the estimates made by the individual panel members. U indicates that the panel member agreed that their individual estimated value was sufficient, i.e., it represents an upper bound. L indicates that it was not. The crossed symbol represents the panel’s consensus estimate. That is, the consensus value is in situation **(A,C)** 0.1 above the highest of the lower estimates in situation **(B)** at the lowest of the upper estimates.

These three scenarios are depicted in Figure 1.

#### Feasibility

To examine the feasibility of this approach, we used it prospectively on cases from the MSF telemedicine network. A panel of observers rated seven cases, which were being assessed routinely for quality assurance purposes.

#### Results

The responses of the panel are summarized in Table 2. The second column contains the answers of each panel member to the question “Considering the teleconsultation as a whole, do you think the quality (value) was sufficiently good in the circumstances? In other words, quality can always be made higher, but was it good enough?” The third column contains, the overall quality score assigned by that panel member, but not revealed to them at the time they answered the question. The fourth column represents, the bound deduced from the panel member’s response, and the fifth column is the estimated value based on the responses from the whole panel.

**Table 2:** **Indirect assessment of case difficulty**.

Case	OK?	GQS	Bound	Target
898	Y	8.6	U	<8.6
914	Y	8.4	U	<8.4
	Y	9.3	U	
	–	9.0	–	
	Y	8.8	U	
	Y	9.4	U	
1201	Y	8.0	U	<6.8
	Y	6.8	U	
	Y	7.5	U	
	Y	7.8	U	
	Y	9.0	U	
1221	Y	8.1	U	6.5
	Y	8.8	U	
	Y	9.1	U	
	N	6.4	L	
	Y	8.1	U	
	Y	8.4	U	
1232	N	6.8	L	6.9
	Y	5.4	U	
	–	6.5	–	
	Y	8.1	U	
1262	Y	8.0	U	7.1
	Y	8.1	U	
	Y	8.7	U	
	N	7.0	L	
	Y	9.4	U	
1290	Y	8.3	U	<6.2
	Y	6.2	U	
	Y	9.0	U	
	Y	9.6	U	
	Y	6.2	U	

Note that the panel estimate was considerably higher in case 914 than in cases 1201 and 1221. This suggests that the latter cases are more “difficult.” Case 914 concerned a request for interpretation of chest X-ray images; this was a relatively straightforward query for the network to handle. Case 1201 was a patient with penile wounds, and case 1221 concerned loss of vision in a patient with multiple drug-resistant TB; both cases can be considered as fairly complicated queries. However, in four of the seven cases, the panel’s estimate was only determined as an upper boundary (e.g., <6.2) rather than a specific value.

### Second method – direct assessment

An alternative method of assessing the difficulty of the question in a teleconsultation network is direct estimation, by having an expert panel rate the difficulty of each case explicitly. That is, suitably qualified observers would independently assess teleconsultation cases by answering the 11 questions about each case shown in Table 3. The scores are then combined by simple summation to produce a rating of difficulty.

**Table 3:** **Direct assessment of case difficulty**.

Question	Response^a^
1. How well formulated was the question?	1 = very poor; 2 = acceptable; 3 = excellent
2. Was the information provided satisfactory? (including, if appropriate, any images and their quality)	1 = no; 2 = perhaps; 3 = yes
3. How severely ill was the patient?	1 = not very; 2 = moderately; 3 = very
4. Were there multiple co-occurring medical conditions?	1 = no; 2 = perhaps; 3 = yes
5. Was it difficult to determine an accurate diagnosis? (e.g., the conditions were poorly differentiated and the symptoms were unrecognized or not identifiable)	1 = not very; 2 = moderately; 3 = very
6. What was the degree of impairment or disability of the patient?	1 = not impaired; 2 = moderate impairment; 3 = very impaired
7. What was the level of need for comprehensive care management?	1 = none; 2 = moderate; 3 = high
8. Was the care-coordinator resource available promptly and with the right experience/expertise to handle the case? (if manual allocation was being used)	1 = no; 2 = perhaps; 3 = yes
9. Was the required specialist(s)/subspecialist(s) available?	1 = no; 2 = perhaps; 3 = yes
10. Did the referral site have satisfactory resources for treatment locally?	1 = no; 2 = perhaps; 3 = yes
11. Was it possible to transfer patients for specialist treatment elsewhere?	1 = no; 2 = perhaps; 3 = yes

*^a^In each case, 0 = do not know was also an acceptable response*.

#### Feasibility

Three observers (experienced telemedicine case-coordinators) independently rated 10 telemedicine cases selected randomly from previous cases in the MSF telemedicine network.

#### Results

**Figure 2:**
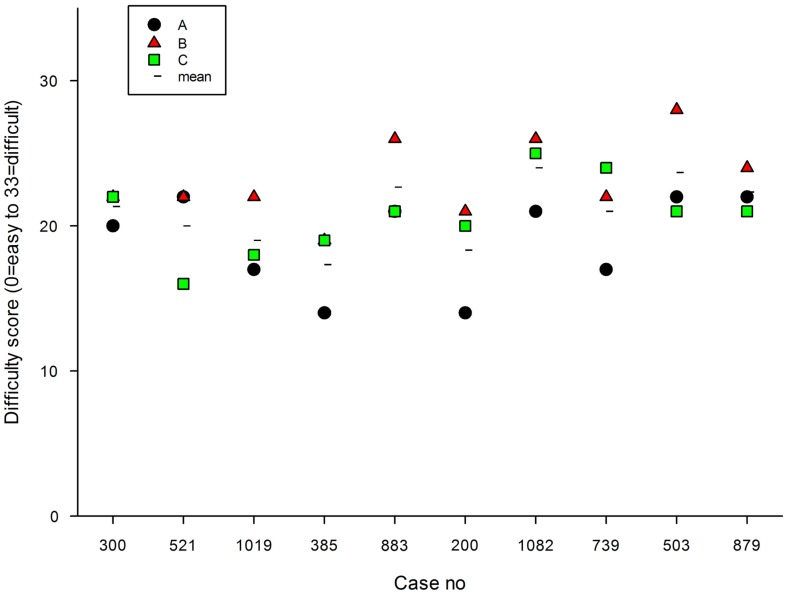
**Difficulty scores in 10 randomly selected cases, rated by 3 observers**. The mean value of the three observers is also shown.

The mean score for difficulty (0 = no difficulty to 33 = extreme difficulty) ranged from 19 (case 1019) to 24 (case 1082), see Figure 2. That is, the difficulty of case 1082 was considered by the panel to be much higher than that of case 1019. Case 1082 concerned a child of 11 months admitted 3 days previously with an unclear history; this could certainly be considered to be a complicated query for the network to handle. Case 1019 concerned the management of a baby aged 5 weeks with an established diagnosis of osteomyelitis; this was a relatively straightforward query for the network to handle.

## Discussion

There are few published reports about quality measurement in telemedicine. Most have been retrospective studies, and concern specific application areas such as radiology (11), ophthalmology (12), or histopathology (13). That is, these reports concern image-based activities, which perhaps lend themselves more readily to quality measurement. In comparison, the situation in teleconsulting is more complex, being inherently multi-specialty in nature and one where there is often limited knowledge of outcomes. Attempting to measure quality in such a context is more like attempting to measure the quality of the consultations taking place in a multi-clinic outpatient department. As far as we are aware, there have been no previous studies of prospective quality measurement in general teleconsulting work in low-income countries. Furthermore, we are unaware of work concerning the differences between cases in a teleconsulting network.

The present work sets out what is required for long-term monitoring of quality in a teleconsulting network. In conventional process control, the output from the telemedicine network would be measured, and compared with a target value. Since it is not straightforward to define the latter, we propose the assessment of the input to the process instead. When each quality measurement of the output is made, an allowance can be made for the characteristics of the case submitted. This means that fluctuations in output quality can be attributed to variations in the process (network) or to variations in the raw materials (queries submitted to the network).

Two methods of estimating the degree of difficulty posed by cases submitted to a telemedicine network have been trialed. The first, an indirect method, is easier to use in practice, but a pilot study shows that it produces results of limited value. The second method, the direct estimation of case difficulty, is more demanding to implement, but produces results, which appear useful. Much further work will be required to develop this method for routine service, so that the individual assessments of case difficulty can be employed in the long-term monitoring of output quality. One simple method would be to normalize the quality score in a particular teleconsultation by dividing it by the difficulty level. However, it cannot automatically be assumed that a linear relationship is appropriate, and a more appropriate weighting scheme might require a logarithmic transformation of the difficulty level. Clearly, these matters all represent areas for future research.

The methodology proposed in the present work is perfectly general, and extends beyond telemedicine in high-resource settings to non-telemedicine work in conventional health care settings. Using a low-resource setting as the environment in which to develop a more general method represents a strength of the study, since it does not depend on a pre-existing, reliable, and efficient health care system to provide a foundation. Long-term quality assurance should assist those providing telemedicine services in low-resource settings to ensure that the services are operated effectively and efficiently, despite the constraints and complexities of the environment.

### Limitations of proposed technique

There are several limitations of the proposed technique (the direct estimation of case difficulty). First, the validity of the method must be established formally. Second, the optimum number of observers remains to be established. Both these matters stem from the sources of variability in the estimation problem being considered, where the underlying true value is obscured by variation between patients, by variation between observers, and also by variation between specialists (although the latter has not been examined previously in the present context).

Finally, the best method of combining the panel’s scores requires some theoretical basis. Clearly, further research is required to investigate all this prospectively.

## Conclusion

As telemedicine becomes adopted as a routine method of healthcare delivery, there is a requirement to implement quality assurance activities. However, there is little published information about quality assurance in store-and-forward networks, especially in low-resource settings. The present study builds on a previous proposal for measuring the quality of individual teleconsultations being produced by a network, and allows long-term process control by taking into account the difficulty posed by individual cases. The methodology is feasible and appears to produce useful results. It should assist those working in low-resource settings to ensure that telemedicine services are operated effectively and efficiently, despite the constraints and complexities of the environment.

## Conflict of Interest Statement

The authors declare that the research was conducted in the absence of any commercial or financial relationships that could be construed as a potential conflict of interest.
